# Roles of molecular neuroimaging techniques in Parkinsonism

**DOI:** 10.15190/d.2023.16

**Published:** 2023-12-31

**Authors:** Emad Singer, Kinal Bhatt, Adarsh Thomas Anthony, Mohamad Badi Dabjan, Sara Muhammad, Jeffrey Tsai, George Michel

**Affiliations:** ^1^The University of Texas MD Anderson Cancer Center, Houston, Texas, USA; ^2^Larkin Community Hospital, South Miami, Florida, USA; ^3^Mayo Clinic, Rochester, Minnesota, USA

**Keywords:** Parkinson's disease, Functional imaging, PET, SPECT, Neurodegenerative diseases.

## Abstract

Parkinson's disease affects millions worldwide and is characterized by alpha-synuclein accumulation and loss of dopaminergic neurons in the brain. Until now, there is no cure for Parkinson's disease, and the existing treatments aim to alleviate symptoms. Parkinson's disease diagnosis is primarily based on clinical observation of bradykinesia, mood, and cognition symptoms. Nonetheless, clinical diagnosis has its drawbacks since symptoms of parkinson's disease only manifest in later stages and can be similar to those of other conditions, such as essential tremors or atypical Parkinsonian syndromes. Molecular imaging techniques, including magnetic resonance imaging (MRI), single-photon emission computed tomography (SPECT), and positron emission tomography (PET), can objectively detect changes in the brain's neurochemical processes and help diagnose and study neurodegenerative diseases. The paper discusses functional imaging objectives, the tracers employed for imaging, and the condition of each target in Parkinson's disease. Functional imaging can bestow invaluable revelations concerning the intricate mechanisms underlying both motor and nonmotor impairments in Parkinson's disease while concurrently illuminating the involvement of striatal dopamine in behavioral phenomena extending beyond mere motor regulation. Furthermore, this cutting-edge technology exhibits great potential in investigating the preclinical stage of the ailment, thereby enhancing our comprehension of the merits and limitations associated with surgical interventions and the efficacy of neuroprotective approaches.

## SUMMARY

1. Introduction

2. Molecular imaging for P.D.

- 2.1 Molecular imaging modalities used in P.D.

- 2.2 Molecular imaging systems and their role in P.D.

3. Implications of functional neuroimaging and molecular targets in different P.D. aspects

- 3.1 Etiology and Pathogenesis3.2 Early P.D.

- 3.3 Correlation with P.D. symptoms

- 3.4 Treatment response

- 3.5 Motor complications

- 3.6 Non-motor dysfunction and complication

- 3.7 Diagnosis

- 3.8 Disease progression

- 3.9 Deep brain stimulation

4. Pros and Cons for each imaging modality in P.D.

5. Discussion

6. Conclusion

## 1. Introduction

Parkinson's disease (P.D.) is considered the second most common neurodegenerative disease, occuring in 0.2% of the global population. Still, this number increases to 1% for individuals aged 60 and over and up to 4% for those aged 80 and over. This indicates a substantial increase in prevalence as people age^^1^^. As the Western population continues to age, the incidence of Parkinson's disease is predicted to increase, resulting in a greater financial load on society due to the high requirement for healthcare resources for individuals with Parkinson's disease^^2,3^^. P.D. is renowned for its manifestation of motor symptoms, including rigidity and tremors. However, patients also experience a range of non-motor symptoms, encompassing cognitive impairment, mood disturbances, gastrointestinal complications, sleep disruptions, and pain^^4-6^^. The combination of these symptoms can vary significantly between patients^^7^^. Unfortunately, there is no known cure for P.D., and present treatments are centered on alleviating symptoms instead of addressing the fundamental cause of the disease^^8^^. 

To diagnose P.D., physicians rely on patient history and clinical examination, including motor symptoms and response to anti-parkinsonism medication, while excluding possible secondary causes^^9,10^^. However, this approach has limitations as P.D. symptoms only become evident at an advanced stage and can overlap with other syndromes like essential tremors or atypical Parkinsonian syndromes^^11^^. Thus, non-invasive imaging modalities such as MRI, Functional MRI (fMRI), SPECT, and P.E.T. have made diagnosing P.D. possible and distinguished it from other conditions in living patients^^12^^. The utilization of molecular imaging in P.D. harbors significant potential in uncovering biomarkers and identifying targets that enable the diagnosis of P.D. prior to the onset of symptoms. Moreover, this approach holds promise in facilitating the development of treatments aimed at addressing the underlying cause of the disease rather than merely alleviating symptoms^^13^^. 

Traditional imaging methods like CT and structural MRI are not very useful for evaluating patients with P.D., as they mainly serve to confirm or rule out other possible diagnoses. Instead, functional imaging techniques are highly effective for assessing functional connections and changes in neurochemistry associated with P.D.. Although these methods are not yet fully established as biomarkers, they can be used to understand the underlying metabolic factors contributing to both motor and nonmotor dysfunction, identify early signs of disease, and monitor its progression. These techniques offer valuable insights into how dopamine impacts reward anticipation, the placebo effect, and other neurobehavioral processes. Our study provides an overview of the available imaging techniques and their potential applications for the study of P.D., emphasizing the importance of functional imaging in this context.

## 2. Molecular imaging for P.D.

Molecular imaging refers to observing, describing, and measuring biological activities at the molecular and cellular levels^^14^^. As a result, molecular imaging can be considered a form of functional imaging because it yields applicable insights into biological processes that occur in vivo^^13^^.

### 2.1. Molecular imaging modalities used in P.D.

There are various molecular imaging techniques used in P.D. patients for different objectives. P.E.T. and SPECT are nuclear imaging techniques that use radioactive tracers to produce images of the tracer's spatial distribution and the distribution of the tracer throughout the body, respectively^^14-16^^. MRI uses strong magnetic fields and electromagnetic pulses to create anatomical images of tissues, and functional MRI (fMRI) detects functional links between brain regions^^17,18^^. These techniques are used in P.D. patients for different purposes, such as aiding in diagnosis and patient selection for clinical studies, evaluating disease progression, identifying differences between P.D. patients and healthy individuals, and assessing treatment efficacy. The use of molecular imaging in P.D. helps achieve these objectives and understand the pathophysiology of the disease.

### 2.2. Molecular imaging systems and their role in P.D.

Molecular imaginguses specialized tracers that specifically bind to molecular targets, such as proteins or neurotransmitters, involved in the P.D. disease process. Labeling these targets with a radioactive or fluorescent marker allows us to visualize their distribution and activity within the brain using various imaging modalities, such as P.E.T., SPECT, and MRI. This allows for non-invasive detection and tracking of disease-related changes in molecular activity over time, which can inform the development of new therapies and help monitor treatment effectiveness. In P.D., molecular targeting has been used to study the role of dopamine transporters and receptors and other neurotransmitters and proteins implicated in the disease pathology. The molecular targets and modality used with each target and their clinical and research applications are summarized in Table 1^^13,19^^.

**Table 1:** The molecular targets and modality used with each target and their clinical and research applications Abbreviations: A.A.D.C, aromatic L-amino acid decarboxylase; P.E.T., positron emission tomography; [18F]F-DOPA, 6-[18F]fluoro-DOPA; [18F]FMT, 6-[18F]fluoro-m-tyrosine; SPECT, single photon emission computed tomography; [11C/18F]DTBZ, [11C](+)dihydrotetrabenazine/[18F] (+)dihydrotetrabenazine; [18F]FEOBV, [18F]fluoroethoxy benzovesamicol; [11C]MeNER, (S,S)-[11C]-2-(α-(2-methoxyphenoxy) benzyl)morpholine; [11C]TMSX, 7-methyl-[11C]-(E )-8-(3,4,5-trimethoxystyryl)1,3,7- trimethylxanthine; DLB, dementia with Lewy bodies; E.T., essential tremor.

A	B	C	D	E
1	Dopaminesynthesis(A.A.D.C)	P.E.T.	[18F] F-DOPA[18F] FMT	Used in practice and research for detecting loss of nigrostriatal dopaminergic nerve endings.[18F] F-DOPA was approved in the EU and the US in 2019 for diagnosing P.D. and distinguishing E.T. from parkinsonian syndromes ^20^
2	Dopaminetransporter	P.E.T., SPECT	P.E.T.:[18F] FE-PE2ISPECT:[123I] FP-β-CIT	In both practical clinical applications and research endeavors, the utilization of this imaging has proven instrumental in detecting the loss of nigrostriatal dopaminergic nerve endings.[123I] FP-β-CIT (DatScan ®) approved in the EU and the US for diagnosing P.D. and distinguishing E.T. from parkinsoniansyndromes ^21^
3	Vesicularmonoaminetransporter	P.E.T.	[11C/18F] DTBZ	In research endeavors, the utilization of this imaging has proven instrumental in detecting the loss of nigrostriatal dopaminergic nerve endings.
4	Dopamine D2/3receptors	P.E.T., SPECT	P.E.T.:[11C] raclopride(+)-[11C] PHNO[18F] fallyprideSPECT:[123I] IBZM	This imaging is extensively employed in research to identify the loss of striatal neurons in multiple system atrophy (MSA) and progressive supranuclear palsy (PSP). It is also utilized for measuring dopamine release and assessing receptor occupancy in various studies.
5	Serotonintransporter(SERT)	P.E.T., SPECT	P.E.T.:[11C] DASB[18F] F-DOPA (off-target binding)SPECT:[123I] FP-CIT (off-target binding)	This imaging is employed in research to effectively detect the loss of serotonergic nerve endings specifically in the raphe nuclei. This approach enables researchers to gain insights into the degeneration of these nerve endings and its implications for various neuropsychiatric disorders and conditions.
6	Serotonin 5-HT1receptors	P.E.T.	5-HT 1A: [11C] WAY100635[18F] MPPF5-HT 1B: [11C]AZ10419369	Only applied in research: loss of 5-HT1R availability found in the cortex
7	Serotonin 5-HT2Areceptors	P.E.T.	[18F] setoperone[11C] Cimbi-36	Only applied in research: changes in 5-HT2AR availability found in P.D.patients with hallucinations
8	Vesicularacetylcholinetransporter(VAChT)	P.E.T., SPECT	P.E.T.: [18F] FEOBVSPECT: [123I] IBVM	In research endeavors, molecular imaging is employed to detect the loss of VAChT specifically in the cortex. This application enables researchers to investigate the implications of VAChT loss in various neurological and neurodegenerative disorders, providing valuable insights into the underlying mechanisms and potential therapeutic targets.
9	Acetylcholineesterase (AChE)	P.E.T.	[11C]MP4A[11C] PMP5-[11C]methoxy donepezil	In research studies, molecular imaging techniques are utilized to detect the loss of AChE specifically in the cortex and the peripheral nervous system
10	Nicotinicacetylcholinereceptors (α4β2)	P.E.T., SPECT	P.E.T.:2-[18F] fluoro-A-85380SPECT:[123I]5-IA-85380	In research investigations, molecular imaging methods are employed to detect the loss of α4β2 receptors throughout the brain. This approach allows researchers to examine the distribution and density of these receptors and investigate their involvement in various neurological and neuropsychiatric disorders.
11	Muscarinicacetylcholinereceptors	P.E.T., SPECT	P.E.T.:[11C] NMPBSPECT:[123I] QNB	In research endeavors, molecular imaging techniques are utilized to detect an increase in muscarinic receptor availability specifically in the cortex
12	Norepinephrinetransporter(NE.T.)	P.E.T.	[11C] MeNER	In research studies, molecular imaging is employed to detect the loss of N.E.T. specifically in the midbrain and thalamus. This application allows researchers to examine the impact of N.E.T. loss in these regions, providing insights into the role of norepinephrine dysfunction in various neurological and neuropsychiatric disorders.
13	Norepinephrinesynthesis inthe heart (NE.T.,VMAT2)	P.E.T., SPECT	P.E.T.:[11C] HEDSPECT:[123I] MIBG	Applied in practice and research: detecting loss of cardiac noradrenergic innervation
14	Synaptic terminals(SV2A)	P.E.T.	[11C] UCB-J	Only applied in research: loss of synaptic terminals found in SN, corticalSynaptic density decreased in P.D. patients with dementia
15	Glucosemetabolism	P.E.T.	[18F] F.D.G	Only applied in research: detecting P.D.-related patterns of metabolism/blood flow/functional connectivity
16	Cerebral blood flow	P.E.T., SPECT,fMRI	P.E.T.:[15O] H2OSPECT:[99mTc] Tc-ECDfMRI: none	In research endeavors, molecular imaging techniques are utilized to diagnose Parkinson's disease and differentiate it from atypical parkinsonian syndromes (APS) at an individual level.
17	Neural connectivity	fMRI	None	
18	Microglia (TSPO)	P.E.T.	(R)-[11C]PK11195[18F] FEPPA	Only applied in research: studying microglia activation in P.D.Elevation of TSPO expression across the brain found in initialstudies, but not confirmed in follow-up studies
19	Adenosine A2Areceptors	P.E.T.	[11C] SCH442416[11C] TMSX[11C] preladenant	Only applied in research: measuring occupancy of A2A targeting drugsIncrease in striatal A2A availability found in P.D. with dyskinesias
20	Cannabinoid CB1receptors	P.E.T.	[18F]MK-9470	Only applied in research: increase in CB1 availability found in the striatum
21	N-methyl-D-aspartatereceptor(NMDA)	P.E.T.	[11C] CNS5161	In research studies, molecular imaging techniques are employed to detect an increase in NMDA availability specifically in the striatum and cortex of P.D. patients with levodopa-induced dyskinesia (LID). By utilizing specific radiotracers that bind to NMDARs, researchers can assess the alterations in NMDAR levels and their potential contribution to the development of LID.
22	Phosphodiesteraseenzymes(P.D.E1-11)	PED	P.D.E4:[11C] rolipramP.D.E10A:[11C] IMA107	Only applied in research: loss of P.D.E found in striatal and cortical regions
23	Neuromelanin	P.E.T.MRI	P.E.T.:[18F] AV1451 (off-target binding)MRI: None	Only applied in research: loss of neuromelanin found in SN and locuscoeruleus (LC)
24	Beta-amyloid	P.E.T.	[11C] PIB[18F] florbetaben[18F] florbetapir[18F] flutametamol	Applied in practice and research: imaging beta-amyloid accumulationThe three 18F-tracers approved for use in A.D. diagnosis in the USand Europe ^22^. DLB patients tend to have higher beta-amyloid load than P.D. patients
25	Tau	P.E.T.	[18F] AV1451[18F] F.D.DNP[18F]MK-6240[18F]PI-2620	[18F] AV1451 and [18F] F.D.DNP are used in practical clinical applications and research studies, molecular imaging techniques are utilized for imaging the load of tau fibrils. By employing specific radiotracers that bind to tau aggregates, researchers and clinicians can visualize and quantify the accumulation of tau pathology in the brain[18F] AV1451 approved in the US for A.D. diagnosis ^23^ PSP patients tend to have higher tau load than P.D. patients [18F]MK-6240 and [18F]PI-2620 are second generation tracers with promising results in identifying atypical P.D. as PSP and corticobasal degeneration ^24^

## 3. Implications of functional neuroimaging and molecular targets in different P.D. aspects 

### 3.1. Etiology and Pathogenesis

The death of cells in the substantia nigra area of the brain can occur many years prior to the appearance of symptoms in P.D. People with genetic forms of P.D. who have not yet displayed symptoms are ideal for studying the preclinical phase^^24-26^^. Dopamine transporter (D.A.T.) SPECT imaging is a well-established technique referenced in scientific literature. It serves as a valuable tool for detecting familial Parkinson's disease and P.D. in first-degree relatives who exhibit a diminished sense of smell. By utilizing radiotracers specific to the D.A.T., this imaging modality allows for the visualization and quantification of dopamine transporter density in the brain^^24-26^^. This imaging method reveals an uneven loss of tracer uptake in the posterior putamen region in the early stages of the illness. Both sporadic and dominantly inherited P.D. exhibit comparable patterns of dopamine dysfunction, with the putamen being more affected than the caudate.Conversely, recessively inherited P.D. affects the caudate to a greater degree. P.E.T. imaging with the benzodiazepine ligand ^^11^^C-PK11195 has been employed to explore the role of inflammation in P.D.^^4^^. The literature revealed increased tracer binding in the midbrain related to disease severity. However, another study found widespread microglia activation outside the brainstem and basal ganglia, with no connection between microglial activation and disease severity or putaminal F-Dopa (F.D.) uptake. A further study used [^^11^^C]-verapamil P.E.T. and discovered that patients with P.D. had increased tracer binding in the midbrain, which may indicate a breakdown in P-glycoprotein function, responsible for removing toxins from the brain^^24-26^^. 

### 3.2. Early P.D.

P.D. symptoms only appear after significant dopamine loss, which equates to about 80% of striatal dopamine content or approximately 50% of nigral dopamine neurons^^24-26^^. This suggests that either the brain has more dopamine than required for daily functioning or, more likely, compensatory mechanisms activate during the pre-symptomatic phase to counterbalance this loss^^24-26^^. P.E.T. imaging of asymptomatic carriers of Leucine-rich repeat kinase 2 (L.R.R.K.2)-associated parkinsonism has demonstrated normal fluorodopa (F.D.) uptake and abnormal dopamine transporter (D.A.T.) binding. These findings suggest an upregulation of aromatic L-amino acid decarboxylase (A.A.D.C) activity and a downregulation of D.A.T. expression. These results align with previous reports highlighting compensatory changes in dopamine processing observed in early sporadic P.D. The P.E.T. imaging technique allows for the non-invasive assessment of dopamine-related molecular changes in asymptomatic L.R.R.K.2 carriers, shedding light on the underlying mechanisms and potential biomarkers associated with the prodromal stages of P.D.^^27,28^^. These changes may explain the focal release of dopamine in the asymptomatic striatum of patients with hemi-parkinsonism; a rare form of P.D. affecting one side of the body^^29^^. Compensatory increases in striatal dopamine turnover occur faster than the reduction in F.D. uptake during early P.D.Along with abnormalities in the nigrostriatal pathway, early P.D. patients also have increased F.D. uptake in the globus pallidus internal segment, possibly reflecting a compensatory alteration^^24-26^^. Postsynaptic D2 receptor binding increases in early, untreated P.D., which may help maintain function despite dopamine deficiency^^24-26^^. Compensatory mechanisms unrelated to dopamine projections may activate during certain tasks, such as motor sequence learning, which involves activations in the cortex beyond those seen in healthy controls, likely compensating for the lack of striatal activation. Asymptomatic carriers of a Parkin mutation exhibit movement-related overactivity in the right rostral cingulate motor cortex and left dorsal premotor cortex during internally selected finger movements, indicating reorganization of motor loops that may suggest pre-symptomatic compensation^^24-26^^.

### 3.3. Correlation with P.D. symptoms

The uptake of Striatal F.D. is a good indicator of bradykinesia, but it does not have a strong correlation with tremors which may be caused by serotonergic dysfunction^^24-26^^. According to research, the P.D.-related metabolic covariance pattern (P.D.R.P.) on F.D.G P.E.T. can be linked to motor disability and disease severity^^24-26^^. P.D.R.P. is characterized by high metabolic activity in the pallidal and thalamic regions but low activity in the lateral premotor cortex, supplementary motor cortex, dorsolateral prefrontal cortex, and parieto-occipital association areas. These findings suggest that the neural circuits responsible for the planning and execution of voluntary movements are involved in this pattern^^24-26^^.

### 3.4. Treatment response

Elevation of the striatal dopamine levels, which can be assessed through Raclopride (RAC) P.E.T. and levodopa, is associated with decreased symptoms of Parkinson's disease. A decrease in P.D.R.P. expression is also linked to a favorable response to levodopa. The subsequent part of this article examines the imaging indicators of motor complications resulting from levodopa and the efficacy of surgical treatments^^24-26^^.

### 3.5. Motor complications

In P.D. patients, loss of dopamine terminals can lead to drug-induced motor complications due to impaired buffering capacity. However, studies suggest that additional mechanisms, such as increased dopamine turnover, may also contribute to motor fluctuations, particularly in younger onset P.D.^^24-26^^. P.E.T. studies using RAC binding have demonstrated an altered pattern of dopamine release in response to levodopa in patients who develop fluctuations, even before clinical evidence is present^^24-26,30^^. The variability in synaptic dopamine levels can cause dramatic changes in receptor occupancy and peak dose dyskinesias. Presynaptic mechanisms contribute to motor complications, while postsynaptic alterations may also play a role in advanced P.D. However, limited imaging evidence supports a correlation between altered dopamine receptors and motor complications. Current imaging techniques are insufficient to assess postsynaptic mechanisms downstream of dopamine receptors, but studies suggest that striatal opioid function may be altered in patients with levodopa-induced dyskinesias. P.E.T. studies have shown altered blood flow in certain brain regions associated with dyskinesias, which could indicate an altered pallidal output to the thalamus and provide insight into the clinical efficacy of pallidotomy as a treatment for these complications^^24-26^^.

### 3.6. Non-motor dysfunction and complication

P.D. is often associated with psychiatric and cognitive complications, possibly due to the degeneration of non-dopaminergic pathways. P.E.T. studies have provided evidence suggesting that depression and anxiety observed in P.D. may be associated with dysfunction in the dopamine and noradrenergic systems within the limbic system. These studies have shown alterations in neurotransmitter activity and receptor binding in regions implicated in emotional regulation, such as the amygdala and prefrontal cortex. Additionally, fluctuations in mood related to levodopa treatment have been linked to dopaminergic modulation of blood flow in the posterior cingulate cortex. These findings highlight the complex interplay between neurotransmitter systems and mood regulation in P.D., providing insights into the underlying neurochemical mechanisms involved in mood disorders associated with the disease. ^^24-26^^. In P.D. patients, cognitive deficits do not respond to levodopa and are treated with cholinesterase inhibitors. P.E.T. studies have also shown that visual hallucinations in P.D. may be associated with relative frontal hypermetabolism and relative hypometabolism in posterior cortical areas on F.D.G P.E.T.^^24-26^^. P.E.T. studies have found that compulsive drug use in P.D. patients may be linked to sensitization of levodopa-induced dopamine release in the ventral striatum, providing direct evidence for the role of dopamine in addiction syndromes. Furthermore, the dopaminergic system may play a role in pain modulation in P.D. P.E.T. studies have demonstrated marked reductions in cardiac sympathetic denervation in P.D. patients, particularly in cases with clinical evidence of autonomic dysfunction. Lastly, REM sleep alteration has been associated with reduced meso-pontine F.D. uptake in P.D. patients^^24-26^^.

### 3.7. Diagnosis

Diagnosing parkinsonism based solely on clinical manifestations may be difficult, especially in the early stages. Functional imaging can be useful in differentiating normal dopamine function from abnormal dopamine innervation, aiding in the early detection of dopamine deficiency, and supporting the diagnosis of atypical Parkinsonian disorders^^24-26^^. However, literature has reported normal imaging in about 10-15% of patients with early P.D., although it is unclear if this reflects insensitivity of the techniques or misdiagnosis^^24-26^^. Additional scans assessing pre- and postsynaptic dopamine function and other imaging techniques like F.D.G P.E.T., diffusion-weighted MRI, and proton magnetic resonance spectroscopy may help improve diagnostic differentiation^^24-26^^. Cardiac scintigraphy can also help distinguish between P.D. with autonomic dysfunction and multiple system atrophy. However, the issue of normal imaging in some patients remains unresolved, and at least some of these patients may have clinical P.D. despite normal imaging^^24-26^^.

### 3.8. Disease progression

Variability in study designs, analytical methods, and the application of different radiotracers makes it challenging to monitor disease progression in P.D. using radiotracer imaging^^24-26^^. Literature has shown a poor correlation between longitudinal changes in P.E.T. or SPECT measurements and clinical progression^^24-26^^. Therefore, imaging measures should not be considered surrogate endpoints in clinical trials of P.D. Despite these limitations, imaging techniques have been applied to estimate the symptomatic threshold and the annual rate of disease progression^^24-26^^. P.E.T. imaging of clinically hemi-parkinsonian subjects suggests that while D.A.T. binding may be the most sensitive tool for detecting dopamine deficiency, it may overestimate the degree of cell loss, particularly in early disease, while F.D. uptake may underestimate the degree of cell loss^^24-26^^. Available P.E.T. data indicate that the level of denervation in the putamen progresses at an annual rate of 5% to 13%, influenced by the stage of the disease and age at onset^^24-26^^. P.D. tends to progress more quickly in early disease, while clinical progression usually tends to be slower in young-onset disease. Recent work shows age-related differences in dopamine turnover, synthesis, and storage in P.D. Patients with younger onset exhibit a greater increase in dopamine turnover relative to the decline in dopamine synthesis and storage. A longitudinal study of asymptomatic individuals with genetic forms of P.D. using multi-tracer P.E.T. imaging may provide more insight into the natural history of the early disease^^24-26^^.

### 3.9. Deep brain stimulation

Pallidal deep brain stimulation (D.B.S.) and subthalamic D.B.S. have both been found to reduce the expression of P.D.R.P., which is related to Parkinson's disease^^24-26^^. However, the mechanisms by which D.B.S. works are not fully understood, and some studies suggest that Sub-thalamic nucleus (STN)-D.B.S. may actually stimulate rather than inhibit STN output neurons. STN-D.B.S. does not increase striatal dopamine release or improve disease progression as measured by a longitudinal decline in F.D. uptake^^24-26^^. STN stimulation affects the frontotemporal network, which may explain some of its neuropsychological side effects. However, designing activation studies is challenging, as changes in behavior across stimulation conditions could affect the results.

## 4. Pros and Cons for each imaging modality in P.D.

Molecular imaging encompasses several key aspects: sensitivity, specificity, accuracy, and temporal and spatial resolution. In preclinical research, SPECT scanners generally exhibit superior spatial resolution compared to preclinical P.E.T. scanners. However, P.E.T. scanners typically offer greater spatial resolution in the clinical setting than clinical SPECT scanners^^31^^. Indeed, P.E.T. generally offers higher sensitivity and temporal resolution compared to SPECT. P.E.T. scanners utilize positron-emitting radiotracers that undergo annihilation events, emitting two photons in opposite directions. This detection of coincidence events allows for accurate localization of the radiotracer and higher sensitivity in detecting and quantifying the radiotracer distribution^^32^^. Hybrid P.E.T./CT or P.E.T./MRI systems offering anatomical reference frames to functional imaging data are more widely available than SPECT/CT and SPECT/MRI systems^^33^^. P.E.T. represents the foremost imaging modality in clinical research, with a significant emphasis on developing novel nuclear imaging agents specifically designed for P.E.T. applications. Notwithstanding its manifold advantages, P.E.T. does exhibit certain limitations, including the requirement for costly scanning equipment and tracers reliant on short-lived isotopes. Consequently, the establishment of P.E.T. imaging centers necessitates close proximity to cyclotron facilities to facilitate the production of these isotopes. In stark contrast, Single-Photon Emission Computed Tomography (SPECT) serves as an older and more cost-effective technique employing longer-lived isotopes, thereby enabling their transportation across substantial distances. Consequently, in imaging centers lacking P.E.T. scanners or the requisite infrastructure to support them, SPECT remains the imaging modality of choice^^34^^.fMRI presents a superior spatial and temporal resolution compared to P.E.T. and SPECT, rendering it a convenient method for quantifying dynamic alterations in neural network activity^^35^^. Moreover, fMRI obviates the need for ionizing radiation, ensuring a non-invasive procedure without contrast agents. Theoretically, there are no inherent constraints on the number of scans that can be performed on a given day. However, it is worth noting that fMRI exhibits a lower signal-to-noise ratio than P.E.T. and SPECT, consequently resulting in a more intricate and uncertain analysis of the imaging data^^36^^.

**Figure 1:**
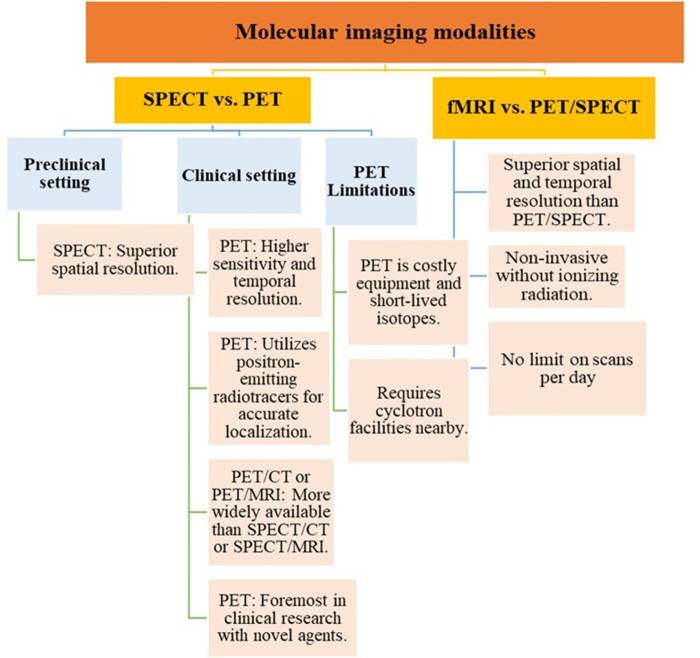
Pros and Cons for different modalities

## 5. Discussion

P.D. is a complex condition that we don't fully understand yet. This makes it challenging to detect and prevent the disease early. Parkinson's disease is diagnosed based on a patient's motor symptoms, with molecular imaging methods serving a supplementary role^^9^^. The significance of molecular imaging in P.D. research cannot be overstated, as it facilitates the investigation of the underlying molecular mechanisms responsible for the onset and progression of the disease in both human patients and animal models. This invaluable capability contributes to the development and evaluation of novel therapeutic interventions for P.D. and holds the potential to enable early diagnosis of the disease prior to the manifestation of symptoms. Molecular imaging studies have unveiled compelling evidence suggesting that P.D. likely represents a collection of related disorders sharing similar clinical manifestations rather than being a singular disease entity^^9,37^^.

The investigation of P.D. through molecular imaging necessitates using specific techniques such as SPECT, P.E.T., and MRI. P.E.T. and SPECT tracers can be tailored to offer valuable insights into molecular targets of interest. Consequently, nuclear imaging techniques play a pivotal role in identifying and characterizing subtle alterations in the brain's molecular architecture associated with P.D.^^13^^. Nuclear imaging techniques such as SPECT and P.E.T. use specific tracers to target molecular features in the brain (as shown in Table 1), including the dopaminergic and noradrenergic systems, which can aid in diagnosing P.D. P.E.T. and fMRI can also be used to study cerebral flow and metabolism, providing diagnostic information^^38,39^^. In order to explore the origins of non-motor symptoms in P.D. and evaluate the efficacy of medications targeting these symptoms, researchers have employed tracers that specifically target receptors and enzymes unrelated to dopamine neurotransmission. By utilizing such tracers, they aim to gain a deeper understanding of the underlying mechanisms contributing to P.D.'s non-motor symptoms and assess the effectiveness of therapeutic interventions designed to alleviate these symptoms^^40-42^^. These studies can ultimately lead to the development of better medications for adjunct therapy of P.D., improving the quality of life for patients.

Despite notable advancements in P.D. research, diagnosis, and treatment, achieving a significant breakthrough necessitates a more profound comprehension of the mechanisms involved in P.D. pathogenesis during the asymptomatic stage. P.E.T. imaging investigations have provided insights by demonstrating that degeneration of dopaminergic connections within the brain initiates several years before the manifestation of P.D. symptoms^^43^^. Nonetheless, a critical requirement exists for tracers capable of targeting the underlying cause of neurodegeneration rather than solely focusing on the observable neurodegenerative changes themselves.

During the asymptomatic phase of P.D., the principal imaging target for unraveling the underlying mechanisms is α-Syn (alpha-synuclein). This protein is widely recognized as the hallmark of P.D., and its aggregation and accumulation within the brain are strongly correlated with neurotoxicity^^44-46^^. Indeed, imaging α-Syn poses a significant challenge. However, researchers are actively working on developing and evaluating various classes of small-molecule probes specifically designed to target and image α-Syn. Despite the complexities involved, these probes have demonstrated promising results in in-vivo studies^^44-46^^.

The imaging of L.R.R.K.2 and mitochondrial complex I present a potential avenue for uncovering the underlying cause of neurodegeneration in P.D. L.R.R.K.2 has been linked to both familial and idiopathic forms of P.D., indicating its significance in the disease process. By imaging L.R.R.K.2, researchers can gain insights into its role and potential contributions to neurodegeneration. Additionally, mitochondrial deficiency, particularly in the context of complex I dysfunction, can lead to increased oxidative stress and neurotoxicity, which are implicated in P.D. pathology^^27,28^^. Histone (de)acetylation represents another potential imaging biomarker in P.D., providing insights into the disease's epigenetic mechanisms. A tracer called [11C] Martinostat has been investigated for imaging histone deacetylase (HDAC) activity and has shown promise in other neurodegenerative disorders^^47^^.

It is crucial to recognize that P.D. may not have a single imaging target to serve as a perfect biomarker^^17,48^^. Indeed, the accumulation of α-Syn is not specific to P.D. alone, as it can also be observed in other neurodegenerative disorders such as dementia with Lewy bodies and progressive supranuclear palsy. This overlapping pathology underscores the need for approaches that can differentiate and characterize these conditions accurately. Multimodal imaging techniques that combine and compare results from different imaging modalities can be valuable in addressing this challenge^^49,50^^.

The development of imaging agents targeting specific suspected targets implicated in P.D. can greatly expedite fundamental research into the mechanisms underlying P.D. pathogenesis. Engineered antibodies capable of active transport across the blood-brain barrier (BBB) hold promise as potential imaging agents in this context^^51-53^^. Preclinical investigations have provided evidence for the efficacy of employing dual-action antibodies that target beta-amyloid in the context of neurodegenerative disorders such as Alzheimer's disease. Moreover, the practicality of these antibodies has been established. They possess the ability to traverse the blood-brain barrier by interacting with transferrin receptors present within the cerebral blood vessels^^54^^. The sluggish movement of antibodies within the body has presented a challenge in the clinical utilization of antibody-based imaging agents. However, promising advancements are being made in the development of pre-targeting strategies that have the potential to overcome this limitation^^55,56^^.

## 6. Conclusion

P.D. diagnosis is based on clinical observation of symptoms, but this has limitations as symptoms only become apparent in later stages and can be similar to other conditions. Molecular imaging techniques such as MRI, SPECT, and P.E.T. can objectively detect changes in neurochemical processes, aiding in diagnosis and study of neurodegenerative diseases. Functional imaging has the potential to provide insights into both motor and nonmotor dysfunction in Parkinson's disease, the role of striatal dopamine in behavioral processes, and the preclinical phase of the disease. This technology may also improve our understanding of surgical interventions and neuroprotective strategies.

## Bullet Points

- Parkinson's disease diagnosis based on clinical observation has limitations due to late-stage symptom manifestation and similarities to other conditions.

- Molecular imaging techniques like MRI, SPECT, and P.E.T. objectively detect neurochemical changes, aiding in diagnosing and studying neurodegenerative diseases.

- Functional imaging offers insights into motor and nonmotor dysfunctions, highlights the role of striatal dopamine, and shows promise in understanding the preclinical phase, guiding surgical interventions, and assessing neuroprotective strategies in Parkinson's disease.
